# BETTER NP PRACTICE ENVIRONMENTS REDUCE HOSPITALIZATION DISPARITIES AMONG DUALLY ENROLLED PATIENTS

**DOI:** 10.1093/geroni/igac059.257

**Published:** 2022-12-20

**Authors:** Jacqueline Nikpour, Heather Brom, Aleigha Mason, Jesse Chittams, Lusine Poghosyan, Margo Brooks Carthon

**Affiliations:** University of Pennsylvania, Philadelphia, Pennsylvania, United States; Villanova University M. Louise Fitzpatrick College of Nursing, Villanova, Pennsylvania, United States; University of Pennsylvania, Philadelphia, Pennsylvania, United States; University of Pennsylvania, Philadelphia, Pennsylvania, United States; Columbia University, New York, New York, United States; University of Pennsylvania, Philadelphia, Pennsylvania, United States

## Abstract

Adults dually-enrolled in Medicare and Medicaid experience twice as many hospitalizations and higher rates of ambulatory care-sensitive conditions (ACSCs) – such as coronary artery disease [CAD] and diabetes, compared to Medicare-only patients. Nurse practitioners (NPs) are well-positioned to address care needs of dually-eligible patients, yet NPs often work in unsupportive clinical practice environments. The purpose of this study was to examine the association between the NP primary care practice environment and disparities in all-cause hospitalizations between dually-eligible and Medicare-only patients with ACSCs. Using linked secondary cross-sectional data from the Nurse Practitioner Primary Care Organizational Climate Questionnaire (NP-PCOCQ) and Medicare claims files, we examined 189,420 patients with CAD and/or diabetes (19.1% dually-eligible, 80.9% Medicare-only), cared for in 470 practices employing NPs across four states (PA, NJ, CA, FL) in 2015. After adjusting for patient and practice characteristics, dually-eligible patients in poor practice environments had the highest odds of being hospitalized compared to their Medicare-only counterparts (OR 1.60, CI: 1.49-1.71). In mixed practice environments, dually-eligible patients had approximately 48% higher odds of a hospitalization (OR 1.48, CI 1.31-1.68), while in the best practice environments, dually-eligible patients had approximately 37% higher odds (OR 1.37, CI 1.21-1.57, p < .001). As policymakers look to improve outcomes and reduce costs among dually-eligible patients, addressing a modifiable aspect of care delivery in NPs’ clinical practice environment is a key opportunity to reduce hospitalization disparities. Yet further efforts are needed to address remaining disparities by meeting patients’ health-related social needs, such as poverty and access to care.

